# ‘TARMACKING’ IN THE MILLENNIUM CITY: SPATIAL AND TEMPORAL TRAJECTORIES OF EMPOWERMENT AND DEVELOPMENT IN KISUMU, KENYA

**DOI:** 10.1017/S0001972013000478

**Published:** 2013-11

**Authors:** Ruth J. Prince

## Abstract

Over the past fifteen years, the city of Kisumu in western Kenya has emerged as an epicentre of ‘global health’ interventions, organized by non-governmental and transnational groups. These interventions involve concrete, practical engagements with the city's populations, but also imaginations and desires, as they intersect with residents’ expectations of development. This article follows the hopes, aspirations and trajectories of people who attach themselves as volunteers to these interventions, or who hope to do so through a process they describe as ‘tarmacking’. In exploring how volunteers orient themselves to ideas of ‘empowerment’ that are promoted by NGOs and also have influence outside institutional settings, it examines the relations between the landscapes of intervention, the spatial-temporal horizons, and the geographies of responsibility emergent in the city. Through its association with ‘moving ahead’ and with development, empowerment implies movement towards some kind of future. While there is a widely shared sense among volunteers that they are going somewhere, just where that might be is not clearly articulated. Rather than attempt to pinpoint this destination, this article follows their trajectories in an attempt to grasp why and how it remains obscure.

## RÉSUMÉ

Au cours des quinze dernières années, la ville de Kisumu dans l'Ouest du Kenya est apparue comme un épicentre d'interventions en « santé mondiale », organisé par des groupes non gouvernementaux et transnationaux. Ces interventions impliquent un engagement concret et pratique avec les populations de la ville, mais aussi avec leurs imaginations et leurs désirs, qui s'entremêlent avec les attentes des résidents en matière de développement. Cet article suit les espoirs, les aspirations et les trajectoires de personnes qui se joignent à ces interventions en tant que travailleurs de santé bénévoles, ou qui espèrent le faire à travers un processus qu'elles désignent par le terme de « tarmacking ». En s'intéressant à la manière dont les bénévoles s'orientent vers des idées d’« autonomisation » qui sont promues par les ONG et ont également une influence en dehors des milieux institutionnels, il examine les relations entre les paysages d'intervention, les horizons spatio-temporels et les géographies de la responsabilité qui émergent dans la ville. À travers son association avec l'idée d’« aller de l'avant » et de développement, l'autonomisation implique un mouvement vers une sorte de futur. Les bénévoles partagent assez largement le sentiment qu'ils vont quelque part, mais sans clairement préciser où se situe ce quelque part. Plutôt que de tenter de localiser cette destination, cet article suit des trajectoires personnelles pour tenter de comprendre pourquoi et comment elle demeure obscure.

I first met Maryon through one of the numerous ‘youth groups’ that have sprouted up in the city of Kisumu in western Kenya during the last decade. With funds secured from a larger ‘youth intervention project’ backed by an international NGO, the group had rented two small rooms in a market street within one of Kisumu's semi-formal settlements and had taken on Maryon as a ‘volunteer’. Wearing heels and a smart trouser suit, her hair elaborately styled, she was sitting at a desk in the office, filling out forms to be taken to the project's headquarters, a large NGO on the other side of the city. She told me that she had finished secondary school three years previously and, after some time living in her parents' rural home and waiting in vain for an opportunity to pursue further education or to get a job, moved to Kisumu to stay with her older sister. The youth group she joined was (at that time) one of the more successful in the city. She was sent on a training week in ‘peer education’ about HIV/AIDS, for which she received a valued certificate. Her task was to ‘mobilize’ young people, to walk around streets and markets and talk to them about HIV. For this, she received 3,000 shillings (about £30) per month. The project did not last long as it ran into funding difficulties, so Maryon found a post as a volunteer ‘youth counsellor’ at another NGO running health education projects in a roadside market outside Kisumu. After this move I lost contact with her and when we met again some months later, she was back in Kisumu, staying with her sister, and ‘tarmacking’, as she put it, looking for work. Unsatisfied with rural life and finding that her volunteering there was not leading to new opportunities, she had returned to the city and, with help from a friend of her sister, began selling second-hand clothes. She had followed her friends to a new youth group, hoping, like them, to become part of a workshop or some further training programme. She had made a CV and was sending out applications for positions as ‘health educator’ or ‘community mobilizer’ for various public health and research projects. Although she had not managed to convert her volunteering or the new knowledge she had gained into employment, Maryon was upbeat about her future. ‘I've been empowered,’ she told me, ‘I am developing myself … I am moving ahead.’

Maryon belongs to a class of aspiring young people who come from families that have enjoyed social and geographical mobility over the past two generations. Their fathers (and sometimes their mothers) were government civil servants – teachers, nurses, railway officers and bureaucrats – until retirement or retrenchment. They thus come from families that were members of rural elites and the urban salariat (termed the ‘working’ classes by East Africans) (Cooper 1983). Many grew up in urban centres and towns and attended boarding schools, and their families had made great efforts to educate them as far as possible. Unlike their parents, however, these young people face no obvious path from education to employment. They came of age during the IMF-backed government retrenchments of the early 1990s and a deepening employment crisis. Government employment provides no openings for them, and a lack of funds prevented many from completing secondary school or college. They thus belong to a generation for whom the promises of modernization associated with the optimistic decades of the 1960s and early 1970s in Kenya have long been out of reach (Haugerud 1993).

The HIV/AIDS epidemic exacerbated and intensified their experience of socio-economic decline. Maryon's generation has lived through the epidemic and their lives have been intimately affected by it. Many lost parents and siblings to AIDS and, as families lost their breadwinners, their own education was disrupted. Yet in the last decade or so HIV/AIDS has also set in motion a new dynamic and new opportunities, as the emergence of Kisumu in the late 1990s as the epicentre of the regional AIDS epidemic attracted international attention and resources and drew in external funds and institutions. A plethora of organizations conducting research on HIV and other diseases operate in the city and its hinterlands, alongside government and non-governmental groups running projects directed at HIV and ‘global health’ (Garrett 2007; Janes and Corbitt 2009).^1^ In 2008, in this city of half a million people, 907 international or national NGOS and local ‘community-based’ groups had registered with the government to run projects ranging from anti-retroviral treatment to orphan care, micro-credit and income generation, health education and ‘the environment’.^2^ In 2006 Kisumu was declared the first ‘millennium’ city, a UN scheme under the patronage of Jeffrey Sachs.^3^ From being a small dusty town bypassed by most labour migrants on their way to other urban centres, Kisumu has become a hive of activity. Fifteen years ago, the occasional government vehicle or minibus taxi could be seen on its pot-holed tarmac roads. Now these are choked by NGO and research project vehicles, by the private cars of its thriving middle class (many of whom work for the NGOs) and by minibus taxis, auto-rickshaws and bicycles.^4^ The Chinese government is building a multi-lane fly-over, and in 2011 the new international airport opened to accommodate the traffic of professionals, consultants, government officials, NGO staff, researchers and business people, both expatriate and Kenyan, into the city. Mega-supermarkets and shopping centres with internet cafes and coffee shops have sprung up alongside new bars, hotels and restaurants, mobile phone shops and adverts for ‘airtime’. All this creates an impression of economic growth (little of which comes from manufacturing and industry) and, if one ignores the simultaneous growth of slum housing (lacking water, electricity or adequate sewage), the promise of entry to a better life.^5^

In the face of earlier disappointments, then, Kisumu appears to Maryon to be a place of opportunity; a space in which, if she avoids slipping through the cracks, she can make something of herself, economically and socially. Through getting ‘exposed’, as she put it, to the city's spaces of intervention, she hopes that she too can place herself in their forward, progressive trajectory. This hope is for economic and social mobility; for a recognized place and position. Yet it is not only about class and self-making. Having grown up in the era of silence and denial about AIDS, many young people like Maryon want to be part of what they see as an opening up of Kisumu and life in it to global languages of human rights and gender equality, to science and ‘positive living’; some want to make Kenya ‘a better place’, others talk of ‘helping the community’. The rhetoric of empowerment draws volunteers like Maryon into HIV interventions in the city and gives them a sense of being able to act, of being part of a forward movement, of *going somewhere*. It connects the mundane work they do to some kind of future. Yet what kind of future that is, and the direction of their movement, remain unclear. Moreover, if one lacks professional qualifications and the right contacts, gaining access to that future through global health projects and NGO work is not easy.

## EXPOSURE, ENLIGHTENMENT AND ‘EMPOWERMENT.’

One way to gain some exposure, if not entry, to this world is to orientate oneself to the knowledge associated with global health projects. In a city focused on the production and circulation of knowledge about health and disease,^6^ narratives about HIV and ‘knowing your (HIV) status’ dominate; they infuse public spaces, are displayed on billboards and posters, are aired on radio and put up as calendars on living-room walls.^7^ They intersect with messages about individual ‘empowerment’ that are promoted by donors, government and NGOs in the move towards ‘community-based development’: by acting on their knowledge, people can ‘take control’ of their health and lives.^8^ Alongside other buzz words such as ‘participation’ and ‘capacity building’, narratives of knowledge and empowerment circulate in the city, fuelling workshops, seminars and training courses through which lay people are encouraged to take part in health interventions. They point to new ways of living in and through the ‘HIV city’, and, as I will explore below, to the possible conversions of knowledge into livelihoods and futures.

The saturation of the city by narratives of empowerment speaks of the narrowing of development horizons towards individual responsibility and self-reliance (Green 2000; Stirrat 2008). Through attending seminars and training sessions and associating themselves with spaces of intervention, volunteers like Maryon seek this empowerment. Yet Maryon's sense of empowerment refers to much more than a narrow version of the responsible subject. It also expresses a connection with a global flow of ideas and discourses, accessed through new encounters and exchanges. Empowerment is closely linked to what people in Kisumu refer to as ‘exposure’ to particular spaces, where they learn certain ways of speaking, knowing and acting, and thus hope to gain a more visible and legitimate presence in the city. As another young woman said of her brother, who after finishing secondary school was working as a cleaner for an HIV clinic: ‘He is getting exposed.’^9^ Exposure can thus be converted into an identity and orientation as an actor and agent in development.

Both ‘empowerment’ and ‘exposure’ are English terms that have no translation into vernaculars – underlining, perhaps, their association with the global. However, they are intimately associated with the broader concept of ‘development’, which has a longer history in Kenya. Development is conceived of as having both a spatial and a temporal orientation; it implies a movement ahead, towards some kind of better future, which relies on spatial connection, exchanges and contacts with elsewhere. Development is also about *being on the move* and it implies a *capacity* to ‘move ahead’ and a sense of purposiveness. The Kiswahili word for development, *maendelelo,* literally means *‘*moving ahead’, as does the Dholuo *dhi nyime*.^10^ These terms imply the presence of both the individual and a collective in a progressive movement forward, and suggest an intimate (if ambiguous) relationship between the two. Development also has a strong ethical element, implying a moralizing relation to others in the cultivation of the ‘proper’ subject.^11^ The sense of development expressed by Maryon through idioms of empowerment and claims to be ‘moving ahead’ resonates with these understandings. At the same time, it departs from a conception of development associated with the immediate post-colonial period, which connected the individual to a clear destination and collective – a modernized nation. Still, even if the destination of their movement remains obscure, people like Maryon share a sense that they are going *somewhere*.

All this suggests that ‘empowerment’ should not be dismissed as the empty or self-deluding rhetoric of neo-liberal development. Instead, the way in which it connects lives, identities and subjectivities to spaces of intervention and global connection should be approached ethnographically. Powerful institutions such as NGOs and government in Kisumu, backed up by international donors, pursue a particular vision of development based upon the empowered individual. Yet in western Kenya development is also intertwined with other histories and trajectories, with continuities as well as departures, which speak to Doreen Massey's (1994) arguments about the multiplicity of space. As a discourse of self-fashioning, empowerment resonates with older trajectories of development in Kenya of both the individual and the collective. Since the early twentieth century, development of both the individual and the collective has been associated with modern education, Western knowledge and science, and thus with a particular vision of the enlightened citizen (Mamdani 1996; Howard Smith 2008; Geissler and Prince 2010). Christianity has been intimately associated with these orientations, through the dichotomy it introduced between past and future, or tradition and modernity (Prince 2007); and through its focus on education as key to the creation of a modern subject (see Peel 1978 for Nigeria). The current association of HIV knowledge with modern ideas about health and education, lifestyle, the body and science is caught up in this historical politics of class, religious identity and difference. In describing herself as ‘enlightened’, Maryon is positioning herself between polarities of progress and tradition, ‘moving ahead’ and ‘backsliding’, which are still resonant today. Meanwhile, associations with movement and purposive direction may relate to older vernacular understandings of well-being and vitality.^12^

Connected to these currents of class and religious identities, references to ‘empowerment’ are not necessarily tied to recent institutional settings; they seep outside them and speak to something beyond them. They can be drawn upon to negotiate intimate relationships as well as public performances. They are used not only by people like Maryon who speak English with ease; women with little formal education pepper their vernacular with ‘*asebedo* empowered’ (‘I have been empowered’). In Kisumu, one hears ‘empowerment’ in the speech of popular Pentecostal preachers who exhort people to develop their sense of self-worth and agency. For those who learn the language of empowerment, then, the HIV city seems to offer a space of anticipation and mobility and a trajectory through which one might ‘move ahead’. But this trajectory intersects with other histories of self-fashioning and class formation as well as with religious (particularly Pentecostal and ‘born-again’) values and orientations. It is through these intersections, perhaps, that empowerment gains deeper meaning and traction in people's lives.

This article uses the prism of ‘empowerment’ to explore the relationships of individuals like Maryon to the work of development – or, as they put it, to ‘moving ahead’. In this approach, I describe both spatial-temporal frames of transformation and the layered geographies of responsibility (Massey 2004) that are being enacted and imagined within and beyond the city. Following the trajectories of street-level workers, volunteers and ‘tarmackers’, I explore how global health interventions in Kisumu are feeding into and shaping imaginaries of development, class and mobility, forms of self-fashioning and attempts to make a successful life (Roitman 2008; Tousignant, this issue). I suggest that in converting these opportunities into possibilities for a future, people may make use of plural orientations, religious as well as secular. While interventions provide some, albeit unstable, opportunities for ‘moving ahead’ through discourses of empowerment and participation, these ways of projecting oneself forward may gain traction through less ephemeral pathways, which relate to a born-again focus on agency, temporality and the self.

I explore these issues through following the trajectories of some volunteers who attach themselves to NGOS, youth groups, HIV clinics and other projects in Kisumu. Taking up Jane Guyer's challenge to explore the temporality of lived economies (Guyer 2011), I try to grasp the forward-moving trajectories and the sense of futures and possibilities expressed by people like Maryon, often in the face of seeming exclusion, frustration and failure. How do interventions in the city engage particular trajectories and imaginations of present, future and past? How do people attempt to make meaningful lives on such terrain?^13^

## ‘VOLUNTEERS’

Volunteers are key figures in official discourses of ‘empowerment’ and their work pervades public health intervention and research in the city, as well as development more generally. It is volunteers who do much of the street-level public health work of ‘mobilizing’ communities, reaching individuals, and providing links between ‘community’ and clinic or project. Volunteers persuade people to come to the clinics or to get an HIV test; they provide counselling; they ‘trace’ people back to their residence and try to keep track of them. In smaller NGOs, they do much of the accounting and auditing work, filling in patients’ forms and updating clinic records. NGOs running projects on orphan care, income generation or home-based care also rely on volunteers, and so do the numerous community-based organizations (CBOs) in Kisumu. Volunteers include people of different ages, social positioning and aspirations. They include middle-aged people with some education who have lost the formal employment upon which they previously relied, widows who have been forced from their rural homes, young aspirational people like Maryon, as well as people who volunteer out of their HIV-positive identity, the ‘poor and positive’ (Boesten 2011). While volunteering was initially more of a female activity (Brown 2013), men are becoming increasingly prominent.

Both the Kenyan government and international donors have promoted the use of volunteers in HIV interventions since the late 1990s, leading to an expansion of opportunities for volunteers to train in and carry out health-related work. This has created a perception that volunteering opens up opportunities for employment in the emerging AIDS economy in Kisumu. While the use of volunteers – part of the trend towards ‘community-based development’ – is widespread beyond health projects (Swidler and Watkins 2009), it has roots in the Alma Ata WHO conference of 1978, with its promotion of community health work and grassroots participation. The shift in Kenya during the past fifteen years towards the use of volunteers is more indebted, however, to the effects of structural adjustment policies, the lack of resources for supporting health systems and professionals, the huge demands that the AIDS epidemic made on the capacities of hollowed-out health systems, the ‘NGO-ization’ of public services (Hearn 1998) and the neo-liberal preference for ‘service delivery on the cheap’ (see Boesten and Mdee 2011: 42).

Figures on volunteers are hard to come by, perhaps because they occupy a grey area between formal and informal work. However, a national survey conducted by the NGO coordination board of registered NGOs cites a figure of 90,411 volunteers who are attached to the 1,334 NGOs that responded to the survey (compare this to the much lower figure of 14,217 officially employed staff).^14^ Actual figures are probably higher, as the survey is based only on NGOs that responded, overlooks much of the work of community-based groups, which are organized by people describing themselves as ‘volunteers’, and does not take into account volunteers attached to government institutions.^15^

These figures emphasize that volunteers are a large but shifting and poorly defined population. While their work is conducted in the formalized space of public health and development interventions, they themselves occupy the ambiguous ground of informal work. Many are taken on through personal contacts and they approach the staff of clinics, projects and NGOs as patrons whose access to training and other opportunities may help them on their way. If successful, particularly in setting up their own CBOs, they may become patrons themselves. Most have no work contract, and while they expect some degree of remuneration, there is no standard to this and many are exploited. Some volunteers receive almost nothing, others receive 3,000 Kenyan shillings a month (about £30), while others are given ‘lunch money’ of 100 shillings for a day's work. Dependent as they are on external funding cycles, projects are time-limited. Many volunteers experience large fluctuations in remuneration, as project funds dry up; some have to wait months for any material benefit and, worn down by waiting, become disillusioned.

Still, many volunteers spend months, even years, volunteering on and off, and since few manage to convert it into an independent livelihood and still fewer into a career, their volunteering must be motivated by more than economic gain. This sense of something beyond a livelihood is captured by Maryon's comment (echoing a common sentiment), ‘I am developing myself.’ Below I consider how ‘exposure’, ‘moving ahead’ and ‘developing oneself’ figure in other volunteers’ trajectories and consider how their visions of development and a successful life may arise from multiple (generational, personal, religious) sources.

## NAVIGATING A POSITIVE IDENTITY

It is early morning, October 2010, in one of Kisumu's fast-growing ‘informal’ settlements. I arrive at Pamela's rented room just after her children (her youngest, teenage son and the three daughters of her deceased sister) have left for school. Pamela is hanging up the curtain dividing her sleeping area from the sitting area of her single rented room, the walls of which are covered with HIV-treatment and education posters and the framed certificates of her attendance at HIV-related workshops. A bucket on the floor collects the drops of water leaking from the roof. She is gloomily contemplating the move she will soon have to make: finding school fees and looking after sick relatives have prevented her from paying her monthly rent (of Ksh800 or £8 a month) for several months, and her landlord wants to kick her out. Gesturing for me to sit on one of the soft chairs, Pamela unwraps some sheets of paper from a plastic bag. These are the ‘follow-up’ forms she is given by the provincial hospital at the start of each week, with the names and locations of tuberculosis patients whom she must visit and observe taking their medicine. I mean to accompany her today, and we set off for the provincial hospital, where Pamela has to deliver her forms and pick up new ones. Together we step carefully over the stream of muddy water and the piles of plastic rubbish that lie embedded in the soil between the rows of hastily constructed houses. Navigating puddles and bicycles, we make our way to the entrance of the hospital, located off one of the city's main road arteries. The dusty pavement is crowded with billboards and hung with banners advertising the upcoming visit of a Pentecostal preacher from Nairobi. A short walk away, an imposingly large cathedral is under construction for one of the many Pentecostal congregations. Under a billboard advertising airtime, Pamela buys twenty shillings (£0.2) of phone credit. A young man in jeans, T-shirt and bandana recognizes her as a community health worker and approaches politely, asking for advice. He says he should have an HIV test; he is worried, but does not know what to do. She tells him she will take him to the hospital's HIV clinic, and the three of us bend our way through the large hole in the wire fence of the compound, the short-cut that saves us walking around to the front gate.

Pamela is a widow who shared the fate of many others in her situation when her husband's family forced her to leave his land, blaming her for his death (Prince 2011). Accompanied by her younger children, she came to Kisumu, staying first with a relative and then, with the little income she got from selling vegetables, in a small room in one of the slums. A neighbour took her to take an HIV test at the Catholic hospital, where she was introduced to a research trial that was providing free anti-retroviral medicines. Through the trial, she joined one of the first HIV-positive ‘support groups’ in Kisumu. She became a vocal witness at ‘mobilizations’, joined other support groups, was invited to workshops, and gained some certificates in HIV work. She became a volunteer in various NGO projects and HIV clinics, providing advice about treatment adherence, visiting ‘clients’, and providing a link between clinic and ‘community’. The remuneration she gains from these activities is as unreliable as other ways of earning an income in Kisumu's informal economy; her current attachment gives her 300 shillings a week, while as a community health worker she was given 3,000 a month.^16^ Through combining her volunteering work with selling vegetables or fried food on the street and with occasional support from her elder son, who works as a mechanic in the informal (*jua kali*) sector, she manages, but only just, to get by.^17^ Through her contacts in NGOs, she got her younger son into a sponsorship programme, which pays his school fees. However, finding enough food for the family is often a problem, and she is often hungry, particularly as the antiretroviral medicines she takes ‘need a lot of food’ (Prince 2012).

Pamela's work with AIDS projects and clinics is not easy. It is difficult to keep track of people who ‘move around’. Some patients stop taking their medication, and, afraid of the disapproval of clinic staff, present themselves as new patients at another clinic. They often disappear from clinic records and cannot be traced, as rental agreements are often informal and people are easily evicted from their rooms. Many do not wish to be identified as HIV-positive, and a visit from someone like Pamela, associated with an NGO or clinic, raises suspicions among neighbours. Having been through similar experiences herself, Pamela is sympathetic. She knows that most young women and mothers, dependent on their male partners for material support, take a great risk if they ‘disclose’ their HIV status, and so may not welcome her visit. Some are suspicious of the motivations for such visits, and accuse her of ‘eating’ the resources to which they believe she has access. At the same time, they may value being connected to her: she can introduce them to staff at the clinics and she can point people towards particular NGOs or research projects that may offer material help or some form of healthcare. Her reputation as a good community health worker depends on being able to negotiate such delicate circumstances (see also Madiega 2011; Madiega *et al*. 2013).

Pamela is under no illusions that her work as a ‘tracer’ or even as a community health worker will lead to employment. She can fill in patient ‘follow-up’ forms but she has little formal schooling (unlike Maryon, she speaks little English and Kiswahili, and is most comfortable in Dholuo). While NGO and clinic staff appreciate her skills at navigating terrain unfamiliar to them – the informal settlements and the mobile lives of those who live in them – there are many like her and, without qualifications, it is difficult to gain lasting recognition. It is in this context that we can understand the value of the numerous certificates that cover the walls of Pamela's room, which attest to her participation in various seminars and workshops. These do not provide her with formal qualifications but they display her exposure to particular spaces – such as the HIV clinics, which, surrounded by prestigious vehicles with NGO logos, embody local, national and global connections, knowledge and skills. They underline her status as a person who is ‘in the know’. The possibilities and limitations of this identity were underlined by the ironic comment made by a member of her HIV-positive support group: ‘We are HIV graduates.’ Volunteering has also given Pamela a sense of connection to something beyond her neighbourhood, and indeed, beyond Kisumu. She once showed me a black bag marked with the logo of an NGO and containing as yet untouched commodities, including soap and a towel. ‘Look at this!’ she joked, ‘I have graduated! I'm at college. And people think I'm studying in America!’

## MAKING CONNECTIONS

Francis is one of the most prominent informal figures who navigate between Kisumu's HIV projects, NGOs, government initiatives and ‘community’ groups. A tall man in his forties, usually dressed casually in a pressed shirt and jeans, with a rucksack over his shoulder and a mobile phone headset, he has been openly HIV-positive since 2000 and is passionate about ‘fighting AIDS in Kenya’. His parents hail from the Rift Valley, but he grew up in Kisumu and speaks fluent Dholuo, Kiswahili and English as well as Kisii, his mother tongue. Francis is a founding member of the first ‘patient support group’ in Kisumu and is on the organizing committee of Kisumu's global AIDS day celebrations. He takes seriously the turn to community development and is passionate about his self-created role as a community spokesperson, acting as a mediator between ‘the community’, government and NGO networks.

Francis has no official position and is not attached to any particular NGO, but uses his contacts to access computers and office space at an NGO office, where, through email and Facebook, he keeps in touch with the many expatriates he meets and with web-based HIV-positive groups. He is forever going to training sessions, enrolling himself in various courses, and gaining certificates and diplomas in a wide array of subjects such as counselling, management and community health. He makes an income partly from the allowances he gets from acting as a representative at workshops and meetings, and from some piecemeal work for NGOs and research projects. His stable income, however, comes from the property his father built in one of Kisumu's informal settlements, a two-storey apartment block, where he and his wife live with their four children and a shifting population of relatives and friends, whom they support. His wife operates a small shop and they rent out the first floor.

Francis grew up in Kisumu. His father worked as a radiologist in the provincial hospital while his mother had her own small business. He spent his childhood in one of the solid middle-class houses built by the Soviets for hospital employees in the 1970s (the main hospital is still known as ‘Russia’), attended a respected Kisumu high school, and went to college. Unable to convert his education into any job, he went through a period of depression, and, as he recalls, began drinking too much while his family suffered. It was through getting sick, finding out and eventually accepting his HIV status, and setting up the patient support group that he pulled himself up. Since then he has built upon and enlarged his network of contacts both within and beyond Kisumu, successfully operating outside formal structures, making connections and seizing opportunities to assert his own role in health projects and ‘community-based development’.

The stories of Pamela and Francis demonstrate that being able to narrate one's life and outlook in terms of a conversion to knowing HIV and responsible living is a vital way through which people attach themselves to global health projects and resources and insert themselves into networks of people and organizations, thereby making a living and even, as they see it (or present it), a new life (see Meinert *et al*. 2009; Nguyen 2010; Marsland 2012; Prince 2012). Pamela and Francis, however, have quite different backgrounds and aspirations, and the differences between them are widening. Pamela has little formal education, spent most of her life in the rural areas and came to the city as a stigmatized woman, separated from her husband and marked with HIV. Francis is a quintessential urban specialist (Blom-Hansen and Verkaaik 2009). While his own trajectory has been precarious, he grew up as part of the urban ‘working’ classes. His parents sent their children to good public schools, invested in land, and built a property in town and a modernized house in their rural home. As volunteering is becoming more competitive, people like Pamela are being replaced by younger or better-educated people like Francis and Maryon, who speak English fluently and have a more ‘professional’ style. And as competition for access to the workshops and the certificates they provide is increasing, people have to be constantly on the lookout for the next opportunity, to reinvent themselves and their skills. The following two vignettes gives us further insights into these lived temporalities of the global health economy and to the importance of style and appearances to mobility and success.

## A ‘WORKING’ IDENTITY

Jomo is a young man in his thirties, who in 2008 was a volunteer for a national NGO in Kisumu. The NGO rented a three-storey building near the central bus stations, where it ran a fee-paying clinic offering ‘reproductive health’ services as well as an HIV testing and counselling room, and it acted as a local anchor for health-related projects funded by donor agencies and international NGOs. Jomo was the ‘director’ of the youth group, and had an office with an old desktop computer on the first floor. He was rarely there, however, as he spent much of his time attending meetings and workshops, conducting training sessions for youth groups, and meeting and socializing with acquaintances, friends and contacts he made among the many organizations in the city.

During our first meeting and for some time after it, nobody told me that Jomo was a volunteer. I was new to Kisumu life, and my impression – reinforced by the fact that he had, or seemed to have, an office and a computer, by his appearance and manner, his leather briefcase and his spectacles – was that he had a paid job. Like other aspiring volunteers, Jomo was immaculately turned out in the style of Kenyan professionals. He always wore an ironed shirt, sober trousers and polished leather shoes. Like Francis, he spoke four languages fluently, and he was busy, always rushing from one appointment, activity, workshop or meeting to another.

It was only much later, when he became frustrated at his failure to convert his position into paid employment, that Jomo admitted to me that he was ‘only a volunteer’. I had called him up while buying some fruit at Jubilee, the lively central market where one could get a cold ‘soda’ (fizzy drink) for 30 shillings (£0.25) and sit in the courtyard on a shady bench. I suggested having a drink together there but Jomo wanted to meet at United, a shiny mall café in one of the new and expensive shopping plazas, which was the hang-out of professionals and, it would seem, the upwardly mobile. A coke at the café there, served by uniformed waiters, costs 150 shillings (at that time, £1.50).

I found Jomo chatting, as was usual, with a knot of acquaintances – he seemed to know everyone in Kisumu. He introduced a middle-aged man to me as the director of counselling at one of Kisumu's colleges: ‘He is the one who helped me so much in life.’ Jomo looked somewhat worried and clearly wanted to talk. ‘Did I tell you the position I have is a volunteer position?’ he began, when we were alone at a table. ‘Even those youth group members don't know I am a volunteer’ – and he explained that, if they knew, he would lose his authority. Like Jomo, many of the volunteers had secondary school certificates, but ‘some have college diplomas, some even have degrees, so many are more learned than I am, even though I know more, I am more experienced’. ‘Why do they volunteer then?’ I asked. ‘To get a reference, to get experience, and some have managed to find jobs, so that encourages others.’ ‘But what about you?’ ‘I am just getting frustrated,’ he replied. ‘I'm just tarmacking.’

Jomo had lost both his parents while still a child. His uncle (FB), who worked as a technician in the state-owned water company, took him and his younger sisters in and supported him through the first two years of a good government secondary school in Kisumu. But he had his own children's fees to pay, and for the next two years of school Jomo was shuffled from relative to relative. With some support from an NGO, he managed to finish school but ‘could not get further’. Finally, in 2006, through his aunt's acquaintance with the director of counselling, he managed to get sponsored by an NGO for a three-week training course – and certificate – in ‘HIV/AIDS counselling’. After further periods of tarmacking, including joining the NGO's youth group, he was taken on as ‘director of youth services’. He received a monthly allowance of 3,000 shillings (£30), and was hopeful that this would translate into a paid position. Unfortunately this period coincided with a decline in the NGO's fortunes – as staff at the head office were accused of misappropriating resources. International donors pulled out, funds were cut and projects slashed, and the period also saw the gradual drift of the youth group volunteers to more successful NGOs.

At the time of our drink at United, Jomo was again sending off his CV to the many organizations working in Kisumu and was hoping to get sponsorship for a part-time course in one of the Kisumu's colleges. He was anxious about his own and his siblings’ futures. His youngest sister had dropped out of school for lack of fees, and then become pregnant. She was staying with their aunt and helping with the housework. In this situation and without a job, Jomo felt unable to move on with his life; he was still sharing a room with a friend and he had a girlfriend but was not planning, as yet, on marriage. He did eventually get sponsorship from an NGO to go on a training course in ‘community development’, and the last time we met he was heading off to Nairobi to take it up.

## CONVERSIONS AND CONTRADICTIONS: TRYING AND TRYING AGAIN

Jomo's story gives some insight into the contradictions of volunteering. Having lost his parents, his education became dependent on rather strained family relations, and, as for so many others, his secondary school certificate proved almost useless. In Kisumu today the few opportunities to ‘move ahead’ and achieve a successful life are bottlenecked around the NGO economy. People attach themselves as volunteers to a project or NGO in the hope of either making a livelihood, like Pamela, or of finding a professional identity and an office job. But they seem rarely to realize these hopes. Instead, these attachments offer much less concrete opportunities: the exposure to new networks, contacts and exchanges, partly composed of local patronage and partly of contacts with globalized spaces, knowledge and skills. The empowerment that people seek appears to reside more in the process of attaching oneself to these spaces, and making use of what one finds there, than in an end result that rarely materializes. The significance of this spatial positioning is demonstrated in the story told by Elias, an age-mate and friend of Jomo. According to Elias, it was the chance he got to sell sodas from a mobile stand located outside an NGO that ‘opened’ a new trajectory, giving him contacts with a network of people and projects, and exposure to an institutional environment. Over a period of five years, through stops and starts, moves forward but also sideways (and often backwards, to the soda stand) he managed eventually to convert this exposure into volunteering, workshops and further training days. His lucky break came when he managed to get one year of paid employment with the NGO as an HIV counsellor.

Mostly, however, volunteers’ trajectories present less a road to success than a struggle back and forth, aptly captured in the idiom of ‘tarmacking’. The reality is that volunteering is more like work in the *jua kali* sector. Like *jua kali* workers, volunteers must work under the hot sun as they traverse the city to find people to ‘mobilize’ or to ‘trace’ clients. Like work in the informal economy, there is no security. The fragility of funds, projects and organizations lends a strong sense of transience to Kisumu's global health interventions. Projects can be closed down and jobs lost in the space of a few months, pushing people who had been employed back into tarmacking or into the informal economy of petty trading, selling sodas, or peddling bicycle taxis. In the five years he spent attached to the NGO, Elias signed up as a clinical trial subject, went to training days, and gained several certificates, but he often found himself back at his soda stand. Another lucky break, a volunteer position for a health project funded by a German organization, came to an abrupt standstill when the donor pulled out. While driving with Elias and other volunteers out of town one day to ‘mobilize’ at a rural school, his boss received a phone call on her mobile, telling her to turn the vehicle round and return to headquarters. By the end of the month the project no longer existed; there were no resources and no payment for the volunteers.

In the face of this insecurity, would-be volunteers attempt to build up their ‘portfolio’ with stacks of certificates, attesting to attendance and skills gained at training days, workshops and coveted courses run by NGOs or colleges. But these attempts are themselves beset with frustrations. Elias tried a computer course until the college running it disappeared overnight, along with his money and certificate. He next tried a course in ‘community health’, but could not keep up with the fees when the project he was volunteering for was slashed. And even certificates quickly become outdated and obsolete, as global health interventions circulate new knowledge and require new forms of expertise.

The constant slippage between the cracks requires a degree of dissimulation, of ‘keeping up appearances’ (see Sadgrove 2007). In a landscape of tenuous projects, unstable flows of donor funds, unreliable colleges and intense competition from others who are struggling to get on, volunteers like Maryon and Jomo put much effort into maintaining a smart, professional look. This contrasts with their residences; many lived in single rooms, shared with friends; some stayed with their relatives; few manage to create independent households or a stable place to live.

Given the inability of families to support further education, fashioning a trajectory also depends a great deal on knowing someone in the network who holds some power and resources. But even then, patronage is unreliable. The willingness of the younger generation of volunteers to speak to me about their lives, and the narratives I was offered, should be understood partly as a search for recognition and also for patronage. Many gave me a well-rehearsed performance, listing their knowledge and skills as if presenting their CVs for a job interview. And many interviews ended with requests for help with college education or employment with an NGO, or with questions about the availability of scholarships and opportunities abroad. Few of the young volunteers ever mentioned their HIV status. Yet they were keen to talk about the obstacles they faced and about their frustrations and anxieties. As we chatted, people told me about the siblings they were proudly supporting or their concerns about getting married and setting up a household. They told of their gratitude to certain patrons, who had introduced them to people or helped them get into a workshop, and of frustrations with others who favoured family members, ‘ate’ or ‘sat’ on resources, or who demanded that they volunteer for nothing while promising eventual employment.

These stories – of trying, trying, and trying again – saturate the lives of the young people who told them. They tell of struggles, both front-stage and back-stage, for recognition and a forward-moving trajectory. Attempts to gain further training, to enrol in courses and to gain employment are stumped by an interrupted or prematurely curtailed education, by a lack of a school-leaving certificate, by lack of contacts or support, and by the transient nature of donor-funded projects and interventions. Yet what is striking about many of the narratives I listened to was their hopeful nature. Like Maryon, Jomo felt that through his exposure to workshops and projects, to being trained and gaining knowledge, he is moving somewhere. Despite repeated setbacks and failures, he keeps trying. Elias talked of how, in spite of his failure for years to get a job, ‘in the process, I got encouraged … I learned a lot’. Faced with the collapse of the project to which he was attached, his response was not doubt but, as he narrated it to me, to ‘look for other options’. The volunteers and would-be volunteers I spoke to insisted that, in spite of their precarious situations, they had been ‘empowered’ and were ‘moving ahead’. This talk of empowerment could, of course, be an artifact of the interview situation; they associated me with the NGO world and they wished to present themselves as competent in a language they thought I could understand – as people who could ‘facilitate’; who had ‘skills and knowledge’ and who were ‘empowered’ (terms often used in interviews). Yet this insistence on empowerment continued in casual conversations, on the street, in people's homes, and throughout longer acquaintances and friendships with some of the volunteers. Hope persisted in spite of frustrations.

Furthermore, there was a strong sense of personal and emotional investment into these precarious trajectories. People drew upon ‘NGO speak’ but they invested it with intense emotions, hopes and aspirations. When Elias received his long-awaited certificate in counselling, based on a part-time course he paid for himself, he told me: ‘I never put it down. I walked with it for almost like … four days, I just admired it and, ah, I could feel proud of myself because … because I had secured one thing that had taken me a long time.’ And in narrating the experience of finally getting his job (the one-year position as HIV counsellor) he became choked with emotion.

E: I took a gospel song and … and … ah *(breaks down)*
R: You took a?
E: A … a … gospel song and ah … sat down and … prayed … and prayed.

Elias's use of gospel songs to express his intense emotions suggests that religious sensibilities and subjectivities may inform both a sense of self and idioms of agency among my informants. Empowerment discourses have a striking resonance with the messages of the popular Pentecostal churches in Kenya. These exhort people to ‘feel your worth’ as a means of overcoming the anxieties of poverty, the loss of loved ones and the precariousness of life. ‘You are valuable!’ preachers tell their congregations. At the Redeemed Gospel Church in Kisumu, sermons last for hours, preachers, pouring with sweat and emotion, bellow into their loudspeakers: ‘Even if you face poverty, even if you face frustrations, even if you have no job and no money, if your business is not doing well, even if your child has died, if you have no children, if you have been frustrated with your education, if your husband does not love you, if people are saying you are useless in your house, even if the devil has messed up your life … you can change this! You can open your heart! You will prosper. You are a great man, you are a great woman!’ Another sermon continued, ‘If you are still sitting here, you have got through what others could not get through. You are sitting here. You have survived! You are victorious!’ These messages project a sense of strength rooted in the self, and of hopeful agency: ‘You can do it!’ While this is clearly connected to a prosperity gospel – ‘You will have a child, you will have a big business, you will become a multi-millionaire!’ – what strikes me here is the focus on the strength of the self, orientated to ‘God's love’ and membership of a church, and ‘manifesting God's will’, as the anchor to a successful life.

Amplified through loudspeakers beyond the confines of the church itself, these messages carry across the city. In Kisumu alone, there are numerous Pentecostal congregations, from large globalized churches broadcasting in Kiswahili and English and appealing to the ‘working’ classes and those aspiring to them (like the Redeemed Gospel, which seats a thousand people) to small offshoots set up by individual preachers speaking vernaculars. Services are filled with emotion, from joy and praise, dancing and singing to tears, physical exhaustion, speaking in tongues and possession. Not confined to official buildings, Pentecostal churches fill the city's public spaces, blasting sermons, prayers and music from loudspeakers erected under temporary sun-shades. Across the city, banners advertise preachers and conventions, mass prayers and meetings. Most of the volunteers I interviewed described themselves as ‘strong Christians’; many were ‘Born-Again’, whose conversations were interspersed with praises to ‘Jesus our Redeemer’. Emails addressed to me with friendly greetings and enquiries about my family are headed with ‘Jesus Saves’, ‘My Redeemer Lives’, and ‘The Lord Is My Strength’. Gospel music is an important part of Christian life and, like Elias, many of my informants found in its messages a way of channelling their own emotions, of cultivating a relationship with God, and, in doing so, orienting themselves to a future that may be uncertain but is guided by the divine. As observed elsewhere, the born-again subject is embedded in relations with a ‘different temporal orientation’, in which the future is much more important than the past (van Dijk 1998:158; Meyer 1998; Simpson 1998, 2003).

## FUTURE DIRECTIONS?

Recent work on African cities highlights the transient, shifting and uncertain nature of urban life today (see, for example, Simone 2004; Mbembe and Nuttall 2004; de Boeck and Plissart 2004). Declining public sector employment, and the privatization and ‘NGO-ization of society’ (Hearn 1998) have led to the disintegration of twentieth-century paths to social mobility and economic security (Banégas 2001; Fourchard 2011). Imaginations of a better future, associated among earlier generations with a faith in modernization and societal development (Parkin 1978; Stichter 1982; Cooper 1983; Burton 2002), have been reduced to the search for a livelihood, amidst the decline of urban infrastructures and the fragmentation of stable frameworks of nationhood and collective progress (Francis 1995; Prince 2005). It is no longer only the urban poor who must struggle to make a living in these environments, but also people who have lost their public-sector or white-collar jobs, and youth with education and aspirations (Ferguson 1999; Durham 2000; Diouf 2003; Christiansen, Utas and Vigh 2005). And while globalized images of consumption, self-fashioning and success dominate urban space in Africa as elsewhere, these hold out the dream of a life that few can lead (Weiss 2002). The present moment is described in terms of ‘crisis’, as scholars analyse the proliferation of fears and moral panics and a growing anxiety about the future (De Boeck 1996; De Boeck and Plissart 2004). For some observers, this uncertainty and instability extends to a ‘crisis’ in post-colonial subjectivity itself (Mbembe and Roitman 1995), as the orientation of selfhood falters on the shifting and insecure terrain of contemporary urban life (Le Marcis 2004).

Like recent ethnographies of work and lives in post-Fordist and post-welfare Europe (Muehlebach 2011; Molé 2011; Muehlebach and Shoshan 2012), this literature points to a rupture in modernity's anticipation of a continuous, forward-projecting time (Masquelier 2002; Carrier 2005). Although these modern times were flawed – in newly independent Africa, the nation and its citizenry were deeply divided and unequal, shaped by colonial racism, embroiled in ethnic and class politics, and led by elites that often treated their less-educated countrymen with contempt (Mamdani 1996) – they were hopeful times, which provided a clear spatial-temporal framework both to people's lives and to political projects (Cooper and Packard 1997; Cooper 2002). Society and nation were conceived of as a whole or at least as an aimed-for political and social collective (Lal 2012). Although this ‘whole’ was never achieved, a meliorist narrative framed development efforts, until undercut by economic and political crisis, structural adjustment and neo-liberalism.

Observations of urban living amongst the debris of earlier periods of optimism resonate with life in Kisumu. In western Kenya, economic contraction and public sector retrenchment overlaid a longer-term decline in rural livelihoods, which the AIDS epidemic brought to a peak (or rather a trough) (Geissler and Prince 2010). Unable to sustain a livelihood on the land, many turned to the city at a time when employment opportunities were contracting. Most struggle to get by and make do in informal ways, selling what they can – small piles of vegetables and fruit, biro pens, second-hand clothes, and fish rejects (in Kiswahili, *mgongo wazi* or ‘open bones’) from the factories exporting to European markets. In recent years, however, Kisumu has developed many of the attributes of globalized enclaves (Ferguson 2006) – pockets of investment, infrastructure and growth – which, in this case, are derived mainly from external donor funds for global health interventions. Bringing resources, expertise and connectivity to a city previously experienced as a backwater, this activity is creating opportunities for residents to access new knowledge, livelihoods and forms of self-fashioning. At the same time, these opportunities are highly circumscribed: bounded to specific interventions, targeting specific populations, and tied to short-term funding cycles and time-limited projects, they produce an ‘archipelago’ pattern of development (Geissler, this issue) rather than a concentric circle of progress. This feeds into growing socio-economic disparities within a ‘city of extremes’ (Murray 2011), as poverty, dispossession and marginalization exist alongside a growing middle class and expatriate populations enjoying globalized patterns of consumption.

It is within these criss-crossing currents of unequal economies, in which economic insecurity and deprivation coexist with bounded enclaves of global funds, connections and lifestyles, that I have described the hopes, aspirations and trajectories of Maryon and other volunteers and ‘tarmackers’ in Kisumu. In doing so, I attend less to the emergence of a new middle class than to those who cannot quite enter it; to the cracks and gaps that appear within and around it and to the wider transformation of life and opportunities beyond formal employment in global health and non-governmental organizations.

Volunteers occupy an ambiguous middle ground here, between those who are actors in a development and global health nexus, and those who are acted upon; between formal employment and informal means of ‘getting by’; and between the disappointed trajectories of previous generations and the hopeful lives to which volunteers aspire. Like many residents of the city, their place is insecure, and they move often, tarmacking in and out of the city, from one rented room to another, and from one project to another. Projects come and go as donors pull out, colleges disappear, and hopes fade of even the insecure employment enjoyed briefly by Elias. The promise of economic and social mobility through empowerment and of inclusion in the NGO world seems ever out of reach (Swidler and Watkins 2009).

For an earlier generation in Kenya, lay participation and voluntary labour in development projects meant being part of the progressive trajectory of the new nation, captured in Kenyatta's call for *harambee* or ‘pulling together’. The failure of such calls for togetherness to translate into development (Howard Smith 2008), disappointment in a politics of the belly (Bayart 1993; Lynch 2011), and the disintegration of government services in the wake of structural adjustment all leave today's volunteers less certain of their social moorings.^18^ The globalized networks of NGOs, transnational research groups, charities, donors and international organizations that have converged on the city do not aim for political change or broad socio-economic improvement, but offer either technical interventions into disease and poverty or a focus on knowledge production and dissemination. They narrow development horizons to the individual, while tying empowerment to time-limited and rather transient projects. Volunteers may strive for empowerment, but the reality is they often spend months or years tarmacking between projects and piecemeal work in the informal economy. Few seem to move far from where they began.

Nevertheless, many of the city's residents regard these interventions as taking the city, and themselves, into a forward-moving trajectory. Projects of global health offer focal points of transformation through which people seek exposure to new ways of speaking, seeing and doing – to new forms of agency and trajectories of self-development. In drawing globalized resources, knowledge, expertise and lifestyles as well as new discourses of gender, health, science and human rights into the city, they materialize hopes for development. Development might be reduced to the individual, and individual trajectories to ‘tarmacking’, but people like Maryon or Elias also feel that they are part of a broader movement forward. They share a sense of being part of an ‘enlightened’ generation; and contrast their lives and attitudes with the ignorance, suffering and fear of the parental generation, whose silence about AIDS marked their childhoods.^19^ ‘We are moving ahead in Kiusmu these days’ and ‘We can do things differently now’ were comments I often heard (and not only from the young and educated). Maryon explained that participation in workshops taught her more than the facts of HIV (which she already knew); it gave her a language to speak about these facts and ability to ‘listen’ and ‘share’. Referring to the death of her own mother from what she suspects to be AIDS-related illness, she commented, ‘If only I knew then what I know now, I could have helped her.’ The rhetoric of empowerment also relates to new ideas about gender relations and women's rights. Such ideas offer a new language and tools for negotiating gender relations. Thus another volunteer – like Pamela, a widow – who had managed to get a paid position as HIV counsellor, proudly told me about being a ‘role model’ for women and about the house she was building, independently of any man, on rural land she had bought for herself and her children. This sense of ‘enhanced agency’ (Moore 2009) is not only about self-making, but encompasses a sense of shared purpose, effort and responsibility, reaching from family relations to ‘helping the community’, and beyond, to a desire to push the country itself forward.

For people like Maryon, then, volunteering seems to open up a field of opportunity in which to fashion a particular subjectivity around an ‘empowered’ self. Their sense of empowerment speaks of a form of self-making that is spatial as well as temporal, where placing oneself within particular spaces becomes a means of orientation and of future growth. Making use of spatial connections and temporal orientations, they place the self at the centre of these efforts (see Diouf 2003; Howard Smith 2008). Perhaps it is because in this context of precarious projects and uncertain economies, broken families and strained relations, the self is the only reliable material of investment (see Feher 2009). If temporal horizons have shortened and people don't really know where they are going, then perhaps the capacity to orientate the self within rapidly changing landscapes is the only option available. But perhaps there are other currents – indicated by the gospel songs and born-again sermons – that are historically resonant and that feed into the fashioning of an empowered self and anchor it as a source of strength.^20^ The fact that the opportunities around ‘global health’ are transient and unreliable, that temporal horizons are limited, and that there is no clear direction, leading to frustrations and disappointments, makes people's hopes of empowerment and the strong sense they express of ‘moving ahead’ even more striking. It suggests that their sense of empowerment cannot be reduced to these externally driven interventions.

We may be sceptical of the Pentecostal call for an ‘enhanced agency’. Yet unlike the ‘crisis’ of the post-colonial subject that Mbembe and Roitman describe, what emerges out of these experiences is a sense of the strength and capacity of the self and its hopeful orientation towards a future (Moore 2009, 2011). Instead of limiting their horizons to the present – as portrayed in the well-worn tropes of urban Africans creatively surviving from day to day – people orientate themselves towards the future (see Guyer 2011). ‘Tarmacking’ evokes just this sense of trying to get by, but it is not simply a survival strategy. It is a search for paths that provide an orientation, that bring one into a spatial and temporal trajectory – one that is, indeed, moving forward, and that places the self and its agency at the heart of development. It is this sense of hope and anticipation of a future that most characterizes people's sense of themselves and their lives.^21^

For the volunteers and tarmackers I have followed here, ‘empowerment’, then, is not only about the self and its development. They feel part of a movement forward. The movement is not clearly defined, perhaps because it is about the opening up of plural trajectories rather than a single narrative of destination. What is clear is that the pouring in of resources and activities into the ‘millennium city’ gives them a sense of being on the map – of being recognized and part of something. Their sense of moving forward arises from their ‘exposure’ to global connections and currents of exchange, and the possibilities these open up. While emerging global health regimes inscribe cartographies of economic, political, cultural and symbolic power onto cities like Kisumu, in engaging with these, people like Maryon, Francis, Pamela, Jomo and Elias bring their own interests to bear, inscribing their own trajectories into the city and, perhaps, opening up future pathways.

## Footnotes

1 US funding has dominated these projects; Since 2004, USAID, which already had a large presence in Kenya (see Hearn 1998), has worked closely with the US PEPFAR fund on HIV-interventions, while the Global Fund and the Gates Fund, as well as the Clinton Foundation and well-known international NGOs such as Care and Action Aid, are present.

2 See the 2007–8 report by the NGOs Coordination Board (set up by the government in 1990): <http://www.ngobureau.or.ke/Publications/National%20Survey%20of%20NGOs%20Report.pdf>, accessed 16 July 2013. It shows that there are 117 NGOs with their headquarters in Kisumu, and 790 operating in Kisumu but with their headquarters elsewhere. See also, for a listing of NGOs working in western Kenya, <http://www.kanco.org/KANCOmembers.php*>,* accessed 16 July 2013.

3 A leading US economist and, prior to his current role in ‘poverty alleviation’, a leading advocate of so-called ‘shock therapy’ to (neo-)liberalize socialist economies.

4 Cf. Pfeiffer 2003, who describes an NGO city in Mozambique.

5 For an exploration of how ‘NGO’ development and its expatriate culture have affected social inequality and space in a Mozambican city, see Pfeiffer 2003. For an account of AIDS and the ‘suffering body’ of the city, see Le Marcis 2004.

6 Public health interventions and research in the city both require and gather data on HIV prevalence levels and infection rates amongst various population groups. The large-scale HIV testing and treatment programmes generate data on numbers reached, while research trials generate knowledge about the effectiveness of particular treatments.

7 Narrating one's life in terms of a ‘conversion narrative’ to ‘positive living’ with HIV has been widespread among those who are ‘poor and positive’, as it is also a means of accessing resources from NGOs (Prince 2012; Boesten 2011). Such narratives are to some extent performative but this does not mean they are insincere.

8 The empowerment of individuals and communities as ‘stakeholders’ is thus both an objective of development and the means of getting there.

9 Wenzel Geissler, personal communication.

10 *Maendeleo* derives from *kuenda*, ‘to go’, and *kuendelea*, ‘to continue’ or ‘go forward’; while *dhi nyime* means ‘to go ahead’. Writing about vernacular concepts of development, both James Howard Smith (2008) and J.D.Y. Peel (1978) observe that among the Taita of Kenya and Yoruba in Nigeria, respectively, an orientation to (external) spaces, and the encounters and mobility therein, have long been conceived as motors of progressive transformation.

11 See Pandian 2008 for a discussion of development as a domain of ethical engagement with the self in south India.

12 The Dholuo substantive for ‘development’, *dongruok*, also means ‘growth’, and its meanings reach beyond development in terms of modern progress and individual advancement, to the well-being, continuity and vitality of families and lineages, homes and households (as well as larger collectives such as lineages and the nation). Rooted in relational practices, the concept of *dongruok* suggests that ‘growth’ is dependent on relationships with others and on the encompassment of individuals by a web of relations, which imbue action with a sense of direction and purpose (Prince 2007; Geissler and Prince 2010).

13 The article is based on ethnographic fieldwork conducted during an extended stay in the city during 2008/9, and subsequent visits in 2010 and 2011. I conducted 42 formal (recorded) interviews and numerous informal conversations with volunteers and with people who described themselves as ‘tarmacking’. I attended the meetings of youth groups, community-based groups, and patient support groups associated with NGOs and HIV clinics operating in Kisumu. I also conducted observations in several HIV clinics and began a long-term study of 20 families with HIV-positive members, most of them living in the informal economy; I shadowed volunteer and community-based workers associated with two clinics and interviewed staff and managers in several NGO and government health centres and HIV clinics in the city and its hinterlands.

14 <http://www.ngobureau.or.ke/Publications/National%20Survey%20of%20NGOs%20Report.pdf>, accessed 16 July 2013.

15 In Kisumu, large numbers of volunteers are linked to the 24 HIV clinics in the city and its immediate surroundings, and these do not appear in the NGO survey.

16 The cost of a 2 kg bag of *ugali* flour, the staple, rose to 120 shillings in 2008, while house rent cost 800 shillings a month.

17 *Jua kali* refers to work ‘in the hot sun’, and refers to all kinds of work in the informal economy, where people must labour under the sun.

18 Stirrat (2008) makes a similar argument in his analysis of the identities and commitments of elite (mostly Western) development workers.

19 For an analysis of the ‘Joseph generation’ in Uganda, see Gusman 2009.

20 The importance of spatial orientations also reveals the relational basis of individual development, which has a longer history in vernacular understandings of ‘growth’. Through attaching themselves to particular spaces, volunteers gain visibility and recognition and are able to cultivate a network of contacts and relationships.

21 This sense of forward progression and hope for the future presents a striking contrast to the ‘precarious’ subjects of European neo-liberal economies (Molé 2011; Muehlebach 2011). Like those who describe themselves as ‘the precariat’ in neo-liberal Italy, volunteers and tarmackers in Kisumu are dependent on unstable, insecure and uncertain economies, in which their own value is easily unrecognized and discarded. Yet, instead of projecting a ‘precarious’ identity, my informants emphasized their hopes for the future and their sense of moving ahead.
